# Current landscape of primary small bowel leiomyosarcoma: cases report and a decade of insights

**DOI:** 10.3389/fonc.2024.1408524

**Published:** 2024-05-23

**Authors:** Junjie Zhou, Houyun Xu, Jibo Hu, Qiang Hong, Xiping Yu, Wei Liu, Jiaxin Zhao, Hongjie Hu

**Affiliations:** ^1^ Department of Radiology, The Fourth Affiliated Hospital, Zhejiang University School of Medicine, Yiwu, Zhejiang, China; ^2^ Department of General Surgery, The Fourth Affiliated Hospital, Zhejiang University School of Medicine, Yiwu, Zhejiang, China; ^3^ Department of Pathology, The Fourth Affiliated Hospital, Zhejiang University School of Medicine, Yiwu, Zhejiang, China; ^4^ Department of Radiology, Sir Run Shaw Hospital, Zhejiang University School of Medicine, Hangzhou, Zhejiang, China

**Keywords:** small bowel, leiomyosarcoma, subtype, preoperative diagnosis, surgical operation

## Abstract

The incidence of leiomyosarcoma (LMS) is about 4–5/100,000 individuals per year. LMSs occurring in the small bowel are even rarer, and their preoperative diagnosis is very difficult. We described two patients with pathologically confirmed small bowel LMS and analyzed their clinical and medical imaging features. Similar cases reported in English in Pubmed database over the past decade were reviewed and summarized. These tumors were categorized by the growth direction and relationship with the intestinal lumen into three types: intraluminal (n = 10), intermural (n = 3), and extraluminal (n = 7). Notably, among the three types of LMS, the intramural leiomyosarcoma stands out as a noteworthy subtype. Emerging evidence suggests that smaller tumor size (< 5 cm) and the intraluminal type may serve as favorable prognostic indicators, while the extraluminal type is associated with relatively poor prognosis. Furthermore, the integration of imaging features with CA125 and LDH biomarkers holds promise for potential diagnostic value in LMS.

## Background

Adult-type soft tissue and visceral sarcomas, excluding gastrointestinal stromal tumors (GISTs), are considered to be rare neoplasms, with an estimated average incidence of 4–5 cases per 100,000 individuals per year in Europe. Leiomyosarcomas (LMSs) have an even lower incidence, with less than 1 case per 100,000 individuals per year (1). The incidence of primary LMS in the GI tract is estimated to be about 1/50 that of GIST (2). The absence of thorough radiological assessments or inaccurate interpretations of image characteristics can result in additional delays in diagnosing primary tumors of the small intestine (3). Nonetheless, patients with small bowel LMS typically present at the emergency department with nonspecific symptoms and insufficient examinations, posing a significant challenge for clinicians in preoperative diagnosis (4–6). The objective of this study was to review our instances of small bowel LMS, including a summary of small bowel LMS cases reported in the past ten years in the English language literature available on Pubmed.

## Cases presentation

**Figure 1:**
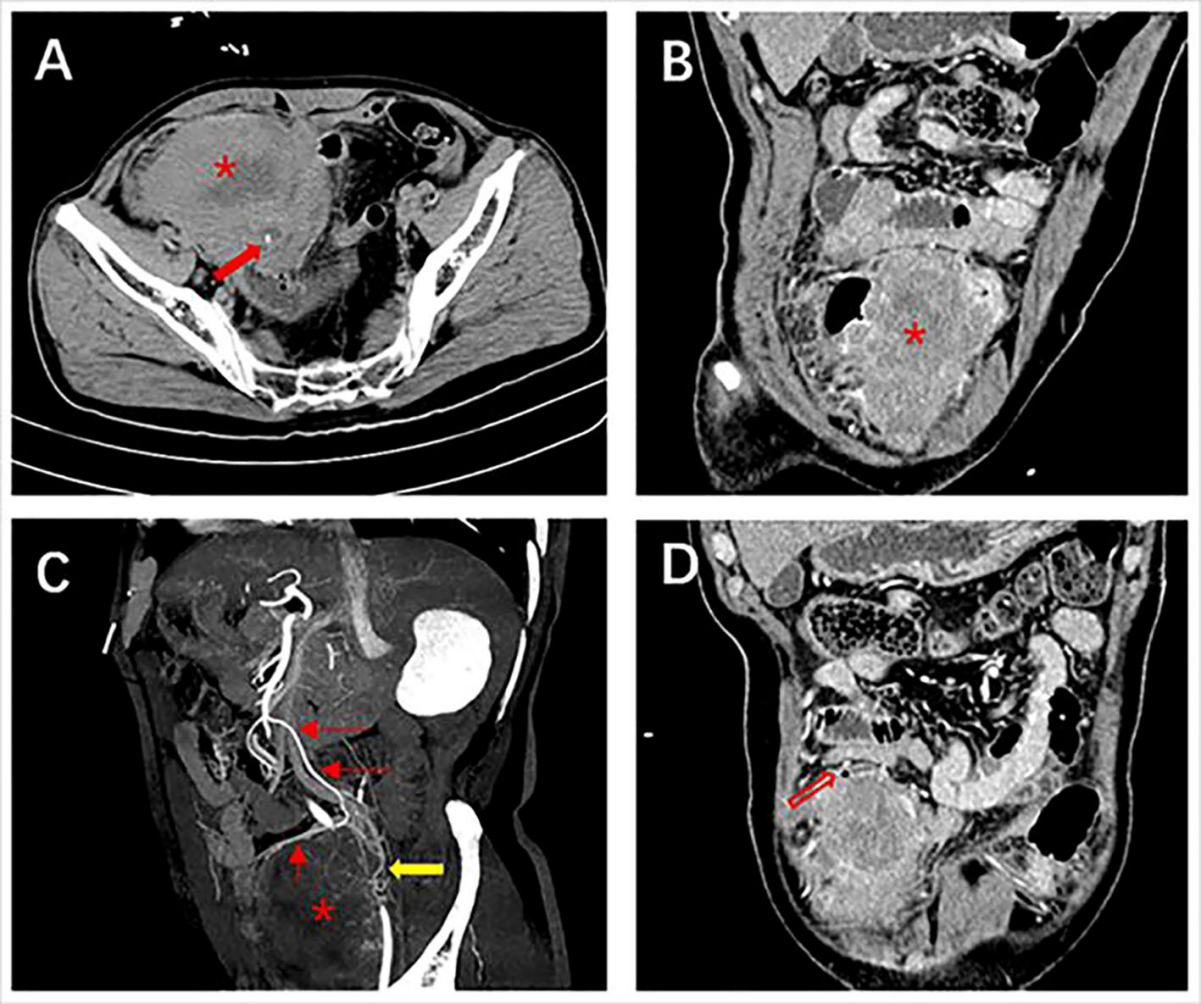
Abdominal contrast-enhanced computed tomography of case one. **(A)** Plain scan shows a large heterogeneous mass (*) with calcification (red solid arrow) at the margins at the end of the ileum measuring 8.7×7.0 cm; **(B)** Adjacent bowels are pushed by the exophytic, heterogeneous enhanced mass; **(C)** The artery supplying blood to the mass is from a branch of the superior mesenteric artery and a tortuous draining vein is seen (yellow arrow); **(D)** Free gas in the abdominal cavity (red hollow arrow).

**Figure 2:**
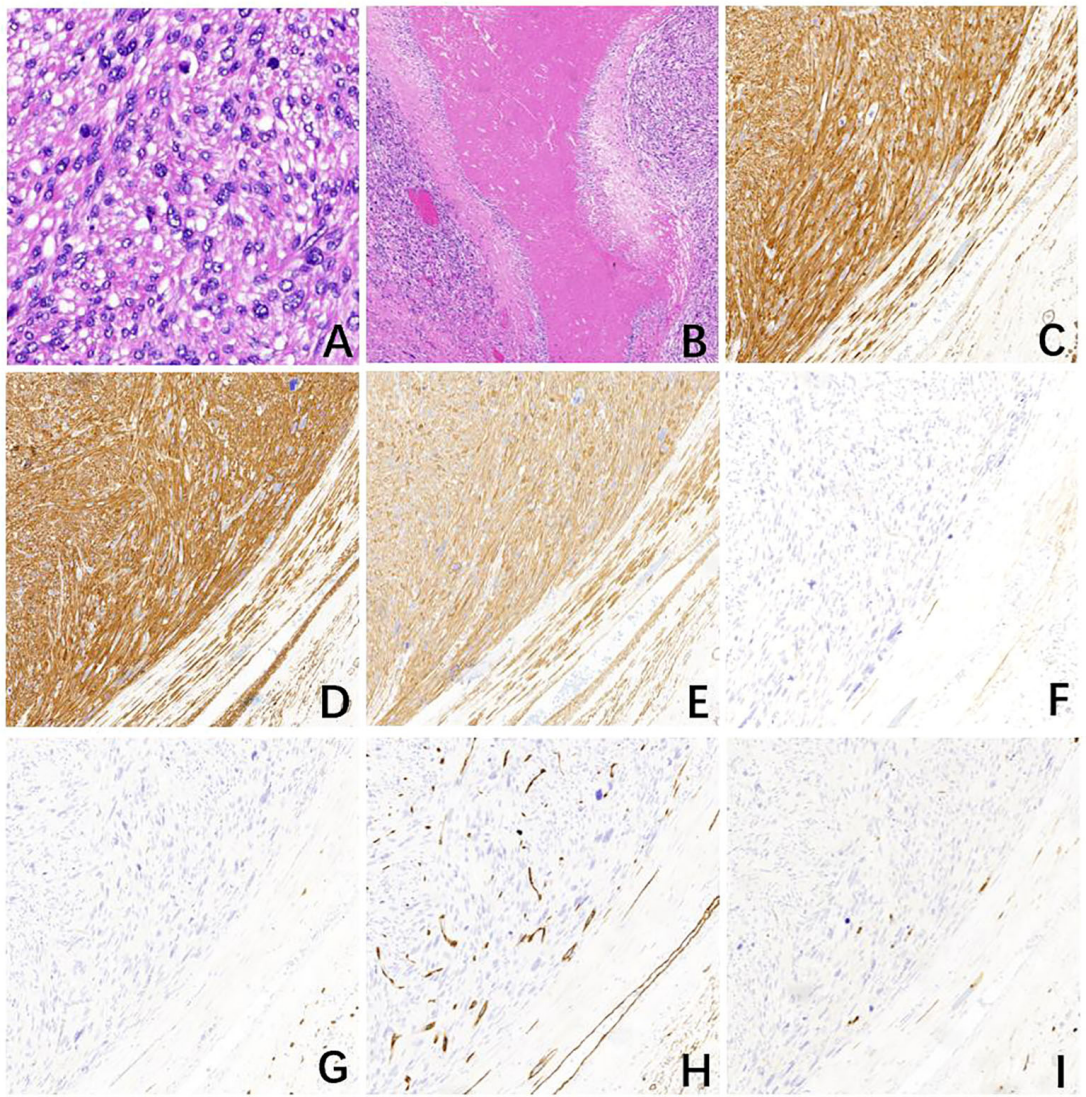
Pathological examination of case one. **(A)** The tumor with mitotically active spindle-shaped cells with severe diffuse atypia and abundant eosinophilic cytoplasm (16 mitoses per 10 high power fields) (H&E, ×400). **(B)** Fibrous septa and large areas of coagulative necrosis within the tumor (H&E, ×40). The tumor cells express **(C)** Desmin, **(D)** smooth muscle actin (SMA), and **(E)** Caldesmon while other markers, **(F)** DOG1, **(G)** CD117, **(H)** CD34, and **(I)** S-100 are negative (H&E, ×100).

*Case 1.* On 5 January 2023, a 70-year-old male patient with a history of hypertension and depression was referred to the emergency department with a complaint of abdominal pain in the lower abdomen which occurred 1 day ago. He denied any other accompanying symptoms like nausea, vomiting, melena, or hematochezia. Over the past several months, he developed a palpable, enlarging abdominal mass. Physical examination demonstrated a palpable mass with tenderness in the right lower abdomen. The patient has a body mass index (BMI) of 19.1 kg/m^2^, and has a smoking history of 40 years, consuming 10 cigarettes per day. He denies any history of alcohol consumption and reports no family history of cancer. A review of the laboratory examination of our patient revealed relevant abnormal indicators as shown in **Table 1**. An abdominal CECT revealed a lobulated, heterogeneously enhancing mass with fibrous separation and extensive necrosis originating from the terminal ileum measuring 8.7×7.0cm (**Figure 1**), accompanied by a blurring of the adipose space and compression of the neighboring intestinal canal. Calcifications were observed at the periphery of the exophytic mass. The CT value of the solid component measured 58 Hounsfield Units (HU) before enhancement and 69 HU during the arterial phase. Maximum intensity projection of axial CECT scan in the arterial phase demonstrates the tumor’s blood supply originating from the superior mesenteric artery. Both radiologists and surgeons concur that the likely diagnosis is GIST. The patient underwent a right hemicolectomy on 10 January 2023, which showed that the tumor was seen on the side of the small bowel in the ileocecum, involving the bladder wall. The mass was adherent to the surrounding small intestine and mesentery. The proximal small intestine was edematous and dilated. Intraoperative blood loss was 100 ml and there were no intraoperative complications. Gross examination showed a large white hemorrhagic lesion with a 0.5 cm-sized perforation (**Supplementary Figure S1**). Histopathological examination showed mitotically active spindle-shaped cells with severe diffuse atypia and abundant eosinophilic cytoplasm (16 mitoses per 10 high power fields) (**Figure 2A**). Fibrous septa and large areas of coagulative necrosis were seen inside the tumor (**Figure 2B**). Immunohistochemical staining was positive for caldesmon, desmin, and smooth muscle actin (SMA), but negative for CD117, DOG1, CD34, and S-100 (**Figures 2C–I**). Finally, the patient has confirmed the diagnosis of low differentiated leiomyosarcoma with negative surgical margins and negative lymph nodes. No lymphovascular or perineural invasion was observed. Ki-67 proliferation index was 50%.

**Table 1:** Review of the laboratory test data of case one.

	Preoperative	F/U 1 month	F/U 2 month	Reference
CA-125 (U/mL)	45.18↑	17.3	14.93	<24.0
CYFRA21-1 (ng/mL)	4.19↑	1.27	1.04	<2.08
NSE (ng/mL)	25.61↑	13.72	13.07	<25.00
LDH (U/L)	306↑	180	191	120-250
D-Dimer (mg/L)	2.5↑	1.89↑	1.93↑	<0.5
CRP (mg/L)	52.1↑	36↑	11↑	0-6

CA-125, carbohydrate antigen 125; CYFRA21-1, cytokeratins 19 fragment antigen21-1; NSE, neuron specific enolase; LDH, lactate dehydrogenase; CRP, C-reactive protein; F/U, follow-up. ↑, higher than reference value.

Postoperatively, he was treated with symptomatic support, and finally discharged without complications on the 15th postoperative day. Epirubicin-based treatment was scheduled by the oncologist. In October 2023, a CECT scan showed a tumor recurrence involving the rectus abdominis muscle. Gastrointestinal perforation occurred during hospitalization and he eventually died of infectious shock. *Case 2. A* 66-year-old man presented with abdominal pain and diarrhea for 7 months. 7 months ago, he was diagnosed with invasive adenocarcinoma lung cancer and received radical surgery. His BMI was 24.1 kg/m^2^. He had been smoking 30 cigarettes per day for the past 40 years, along with consuming 200 ml of white wine daily for 30 years. He denied any family history of cancer. Before this admission, he had undergone abdominal CECT which found a 4.8×4.2 cm tumor originating from the horizontal part of the duodenum. Laboratory examinations showed all results within normal limits except for cytokeratins 19 fragment antigen21–1 2.11 ng/mL (normal range < 2.08 ng/mL). The patient underwent segmental duodenal resection on March 20, 2023, with intraoperative blood loss of 20 ml. There were no intraoperative or postoperative complications, and the patient was discharged on the 7^th^ postoperative day. Histology confirmed that the tumor was immunohistochemically positive for SMA, and desmin, while CD34, CD117, and DOG1 were negative (**Supplementary Figure S2**). The final diagnosis of the tumor was a well-differentiated duodenal LMS. Ki-67 proliferation index was 30%. Surgical margins and lymph nodes were negative. No indications of lymphovascular or perineural invasion detected. There were no significant signs of recurrence in the operated area and other organs on abdominal CECT in the 7-month follow-up.

## Discussion

To gain a comprehensive understanding of small bowel LMSs from various perspectives, we have compiled the characteristics of patients reported in the last decade, focusing solely on English language literature available on Pubmed. Based on the growth direction and relationship with the intestinal lumen, the LMSs can be categorized into three types: a) intraluminal, characterized by predominant luminal growth patterns; b) extraluminal, where growth extends beyond the intestinal lumen into adjacent tissues; and c) intramural, which exhibits infiltrative growth along the intestinal wall, resulting in thickening of the intestinal wall as the primary manifestation. **Table 2** provides a comprehensive overview of the last decade reported cases of small bowel LMS (n = 20), categorized by survival duration. The age ranged from 38 to 90 years, with a median of 69.5 years. Of the total cases, 16 were male and 6 were female. The most prevalent symptom was abdominal pain, followed by weight loss, intestinal obstruction, gastrointestinal bleeding, and anemia. Additionally, non-specific gastrointestinal symptoms such as nausea and vomiting may manifest in the early stages of the disease. Among the identified tumors, 2 were located in the duodenum, 5 in the jejunum, 1 in the duodenal-jejunal junction, and 12 in the ileum. 2 of which have no site of origin mentioned. The average size of the tumors was 8.2 cm (range, 1.6–15 cm). A lobulated heterogeneous mass was the most common appearance in imaging findings, followed by intussusception, perforation, calcification, and thickened intestinal walls. Most of the cases underwent surgical resection, out of 16 patients with available data, 37.5% (6/16) underwent laparoscopic surgery while 62.5% (10/16) underwent laparotomy surgery. Mitotic figures ranged from 2 to 50 per 10 HPFs. SMA, desmin, and caldesmon are positive in LMSs at most times, while other markers CD117, CD34, DOG1, S-100 are negative. A total of 20 patients were grossly typed in all 22 cases (Intraluminal = 10; Intramural = 3; Extraluminal = 7). Statistical analysis of the characteristics between the different types is shown in **Supplementary Table S1**. Among the 6 patients who survived for 12 months or longer, gross assessment was conducted on 5 of them. 4 out of the 5 patients (4/5) had intraluminal tumors, and 3 out of the 4 patients (3/4) remained disease-free at the end of the follow-up period. The average tumor size among the five patients was 5.28 cm. Among the 10 patients with a survival time of less than 12 months, 9 were available for gross assessment. Out of these 9 patients, 4 of them had intraluminal tumors (4/9), and one died from a different disease while the others showed no evidence of disease. Among the remaining 5 cases with an extraluminal growth pattern, three patients had died, and the average size of the extraluminal tumors was 10.8 cm. Despite the small bowel comprising 75% of the length and 90% of the absorptive mucosal surface of the gastrointestinal tract, the occurrence of malignant tumors originating from the small intestine is less than 5% (24, 25). Our study revealed a higher prevalence of primary small bowel LMSs in males, particularly in their sixties. These LMSs commonly manifest as an acute abdomen, resulting from complications such as intussusception, intestinal obstruction, and GI bleeding. Consequently, patients with LMSs often require admission to the emergency department. Among the small bowe segments, the ileum exhibits the highest incidence of LMSs, followed by the jejunum and duodenum. Interestingly, our study revealed that, in addition to extraluminal and intraluminal growth patterns, the intramural type of growth has also been documented as diffuse thickening of the intestinal wall, resembling the aneurysmal dilatation that is a characteristic imaging feature of lymphoma. The smooth muscle cells originating from the muscularis mucosa or propria, which are responsible for the development of LMSs, typically consist of elongated cells. (26) This observation may explain the lymphoma-like behavior exhibited by LMSs. In cases where the tumor exhibits a mitotic activity exceeding 10 per 10 high-power fields, the presence of invasive behavior becomes apparent (27). However, our study is currently unable to provide substantial support for this perspective due to limitations in available information. Despite the scarcity of clinical data, the observed trend suggests that smaller tumor size (< 5 cm) and the intraluminal type may serve as favorable prognostic factors. Regrettably, our evaluation did not encompass intramural LMSs. In the first case, a patient was admitted due to the manifestation of paroxysmal abdominal pain exclusively. Upon postoperative re-examination of the images, a minute presence of free gas within the abdominal cavity was observed (**Figure 1D**). To the best of our knowledge, the intestinal perforation observed in this particular case represents the fourth reported instance since the World Health Organization’s refinement of LMS (7, 18, 28). In the second case, the patient initially received treatment for lung cancer, and his laboratory tests indicate relatively normal results. The final diagnosis of LMS of the duodenum may have been fortuitous. The detection rates of LMSs are relatively low when using colonoscopy and esophagogastroduodenoscopy (16). Our current understanding of the imaging characteristics of LMS is limited due to the rarity of observing small bowel LMSs and the inadequacy of examinations. However, abdominal CECT still proves to be relatively effective in identifying lesions, although distinguishing between LMSs and GISTs can be challenging. A size exceeding 6 cm and the existence of irregular margins, with or without peripheral lymphadenopathy, indicate a considerable likelihood of malignancy, specifically either a GIST or the less common LMS (29). Goto et al. have mentioned that the combination of contrast-enhanced dynamic MRI with serum LDH levels has demonstrated a specificity, positive predictive value, negative predictive value, and diagnostic accuracy of 100% in distinguishing uterine leiomyosarcomas from degenerated leiomyomas (30). Zak et al. conducted a comprehensive review of multiple studies investigating the potential correlation between clinical indicators, including D-dimer, CRP, CA125, LDH, among others, and the presence of uterine LMSs for preoperative identification (31). However, it is important to note that abnormal tumor markers are relatively infrequent in small bowel LMSs (15–17). Furthermore, there is currently no consensus regarding the role of CA125 in the diagnosis of LMSs. Nevertheless, when combined with imaging features, elevated levels of serum CA125 and LDH may contribute to the accurate diagnosis of LMSs. In our case, an MRI was not performed due to the emergency department procedure, however, the CECT in combination with laboratory indicators may have a potential diagnostic value of LMSs. Given the rarity of small bowel LMS, the absence of specific guidelines necessitates that surgical excision with negative margins (R0) remains the primary treatment objective (7). LMSs infrequently exhibit lymph node metastases, thus advanced lymph node dissection is typically unnecessary during these procedures (10). It is noteworthy that abdominal wall muscle involvement was detected in the three patients who experienced tumor recurrence, and laparotomy was employed as the surgical approach for all three patients (No1, 8; our case 1). Laparoscopic resection, which is less invasive, is considered a viable alternative to laparotomy (10). Neoadjuvant and adjuvant chemotherapy may be considered the preferred treatment option following a personalized multidisciplinary assessment, despite the lack of established efficacy and general recommendation (1).

**Table 2:** Summary of small bowel leiomyosarcoma over the past decade reported cases arranged by length of survival.

No.	Age/sex	Clinical manifestations	Site	Size (cm)	Imaging findings	Surgery	Gross	Mitoses/10 HPFs	Immunohistochemistry	Outcome	Survival (mo)
1 (7)	79y/F	Intestinal obstruction	Ileum	NM	Recurrence (Ileum, colon, and abdominis muscle)	NM	UE	NM	NM	TR	64
2 (8)	38y/M	GI bleed, weight loss	Duodenum	1.6	Ulcerative hypodense lesion	Laparotomy	Intraluminal	21	SMA+, desmin+, CD117-, CD34-, DOG1-, S-100-	ANED	32
3 (7)	83y/F	Abd pain, intestinal obstruction	Jejunum	5	Mass and jejunal invagination	Laparoscopy	Intraluminal	NM	SMA+, caldesmon+, desmin+, CD117-, CD34-, S100-	DOD	15
4 (9)	54y/M	Anemia	Jejunum	10	Intussusception	Laparoscopy	Intraluminal	>20	SMA+, caldesmon+, CD117-, CD34-, DOG1-, S-100-	ANED	12
5 (10)	87y/M	Abd pain, weight loss	Jejunum	5	No evidence of tumor	Laparoscopy	Intraluminal	28	SMA+, CD117-, CD34-, S100-	ANED	12
6 (11)	80y/M	Abd pain, anemia	Ileum	4.8	Intussusception	Laparotomy	Intraluminal	NM	Caldesmon+, desmin+, CD117-, DOG1-	ANED	12
7 (7)	86y/M	Intestinal obstruction	Ileum	12	Pulmonary and mesenteric root metastasis	NP	Extraluminal	NM	SMA+, caldesmon+, desmin+, CD117-, CD34-, S100	DDD	11
8 (7)	69y/M	Peritonitis	Ileum	NM	Recurrence (Ileum, caecum)	NM	UE	NM	NM	TR	7
9 (12)	45y/F	Abd pain	Ileum	8	Heterogeneous mass	Laparoscopy	Intraluminal	2-4	SMA+, desmin+, CD117-, CD34-	ANED	6
10 (13)	65y/M	Abd pain	Ileum	6.5	Lobulated heterogeneous mass	Laparotomy	Extraluminal	NM	Caldesmon+, desmin+, CD117-, DOG1-	ANED	4
11 (14)	80y/M	Abd pain	Ileum	3.5	Intussusception	Laparoscopy	Intraluminal	40	SMA+, desmin+, CD117-, DOG1-, S-100-	DDD	3
12 (15)	43y/F	Peritonitis	Ileum	14.7	Perforation	Laparotomy	Extraluminal	40-50	SMA+, desmin+, CD117-, CD34-, DOG1-, S-100-	ANED	2
13 (16)	67y/M	Distended veins, weight loss	NM	12	Lobulated heterogeneous mass Hepatic masses	NP	Extraluminal	NM	SMA+, desmin+, CD117-, DOG1-	DOD	2
14 (17)	90y/M	Abd pain	Ileum	13	Intussusception	Laparotomy	Intraluminal	NM	SMA+, desmin+, CD117-, CD34-, DOG1-, S-100-	ANED	NM
15 (18)	66y/M	Abd pain	Jejunum	15	Perforation	Laparotomy	Intramural	NM	SMA+, desmin+, CD117-, CD34-, DOG1-, S-100-	NM	NM
16 (19)	62y/F	GI bleed	Ileum	9	Exophytic mass with calcifications	Laparotomy	Extraluminal	5-10	SMA+, caldesmon+, desmin+, CD117-, CD34-, DOG1-, S-100-	ANED	NM
17 (20)	72y/M	Abd pain, weight loss, and GI bleed	Jejunum	7	Thickened intestinal wall	NM	Intramural	8	SMA+, desmin+, CD117-, CD34-, DOG1-	NM	NM
18 (21)	72y/M	Abd pain	DJJ	7	Asymmetric luminal dilation and wall thickening	NM	Intramural	NM	SMA+, caldesmon+, desmin+, CD117-, CD34-, DOG1-, S-100-	NM	NM
19 (22)	45y/F	Abd pain	Ileum	NM	Intussusception	Laparotomy	Intraluminal	34	SMA+, desmin+	NM	NM
20 (23)	83y/M	Anemia	NM	NM	Lobulated heterogeneous mass	Laparotomy	Extraluminal	NM	SMA+, caldesmon+, desmin+, CD117-, CD34-, S100-	ANED	NM
21	70y/M	Abd pain	Ileum	8.7	Perforation and calcification	Laparotomy	Extraluminal	16	SMA+, caldesmon+, desmin+, CD117-, CD34-, DOG1-, S-100-	DOD	9
22	66y/M	Abd pain	Duodenum	4.8	Heterogeneous mass	Laparoscopy	Intraluminal	7-8	SMA+, desmin+, CD117-, CD34-, DOG1-	ANED	7

ANED, alive, no evidence of disease; DDD, dead of different disease; DJJ, duodenal–jejunal junction; DOD, dead of disease; NM, not mentioned; NP, not performed; SMA, smooth muscle actin; TR, tumor recurrence; UE, unable to evaluate.

## Conclusion

Based on our comprehensive literature review and extensive experience, the classification of LMS encompasses three distinct types: intraluminal, intermural, and extraluminal. Notably, intramural leiomyosarcoma stands out as a noteworthy subtype. Emerging evidence suggests that smaller tumor size (< 5 cm) and the intraluminal type may serve as favorable prognostic indicators, while the extraluminal type is associated with relatively poor prognosis. Furthermore, the integration of imaging features with CA125 and LDH biomarkers holds promise for potential diagnostic value in LMS.

## Data availability statement

The original contributions presented in the study are included in the article/**Supplementary Material**, further inquiries can be directed to the corresponding author/s.

## Ethics statement

The studies involving humans were approved by The Ethics Committee of The Fourth Affiliated Hospital, Zhejiang University School of Medicine. The studies were conducted in accordance with the local legislation and institutional requirements. Written informed consent for participation was not required from the participants or the participants’ legal guardians/next of kin in accordance with the national legislation and institutional requirements. Written informed consent was obtained from the individual(s) for the publication of any potentially identifiable images or data included in this article.

## Author contributions

JuZ: Writing – original draft, Writing – review & editing. HX: Writing – original draft, Writing – review & editing. JH: Writing – original draft, Writing – review & editing, Conceptualization, Supervision. QH: Writing – original draft, Writing – review & editing. XY: Writing – original draft, Writing – review & editing. WL: Writing – original draft, Writing – review & editing. JiZ: Writing – original draft, Writing – review & editing. HH: Writing – original draft, Writing – review & editing.
