# Supplementary material for: Current landscape of primary small bowel leiomyosarcoma: cases report and a decade of insights
Source: Front Oncol. 2024 May 23;14:1408524. doi: 10.3389/fonc.2024.1408524 (PMC11153743; doi:10.3389/fonc.2024.1408524)
Supplement: Supplementary file 1 [file DataSheet_1.docx]

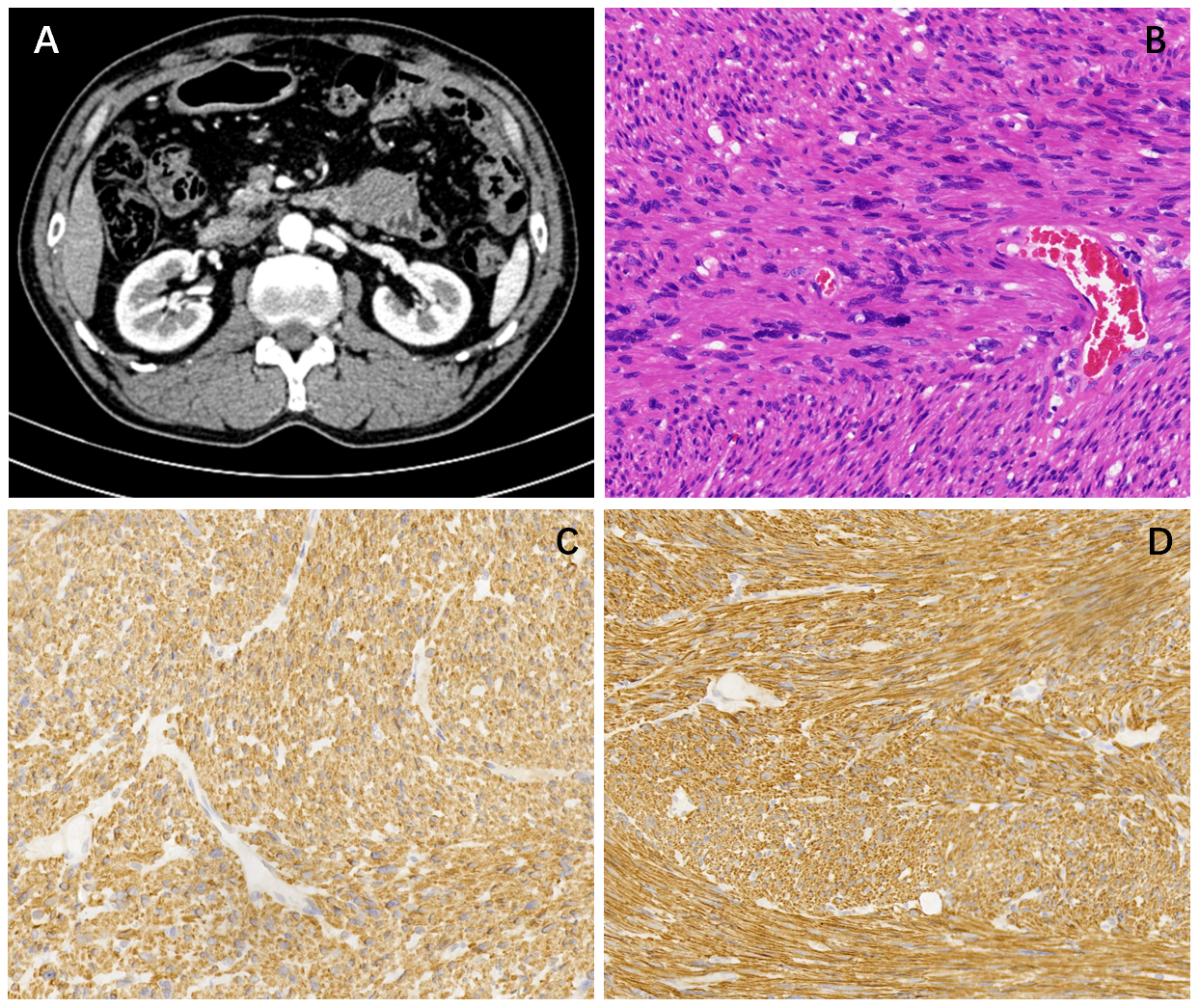
**
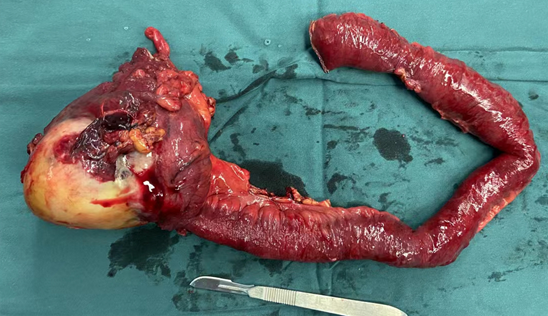
Figure S1.** Gross photograph of resection specimen showing the ileal leiomyosarcoma of case one.

**Figure S2.** (A) Axial CT showed that the tumor was closely related to the duodenum. (B) H&E, ×400. (C) Desmin. (D) smooth muscle actin (SMA).

**Table S1.** Different characteristics between the three types of LMSs.

|  | Gross | | | *P* value |
| --- | --- | --- | --- | --- |
|  | Intraluminal (n = 10) | Intramural (n = 3) | Extraluminal (n = 7) |  |
| Age (y) | 67 ± 20 | 70 ± 3 | 68 ± 14 | 0.962 |
| Male (n) | 7 (70%) | 3 (100%) | 5 (71%) | 0.093 |
| Site (n)^a^ |  |  |  | **0.037** |
| Duodenum | 2 (20%) | 0 | 0 |  |
| Jejunum | 3 (30%) | 2 (67%) | 0 |  |
| Ileum | 5 (50%) | 0 | 5 (100%) |  |
| DJJ | 0 | 1 (33%) | 0 |  |
| Size (cm)^b^ | 6.2 ± 3.5 | 9.7 ± 4.6 | 10.5 ± 3.0 | 0.076 |
| Survival (mo)^c^ | 11.1 ±9.0 | UM | 5.6 ± 4.2 | 0.230 |

^a^Missing in 2 patients. ^b^Missing in 4 patients. ^c^Missing in 6 patients. DJJ, duodenal–jejunal junction.
